# Age-Dependent Loss of Mitochondrial Function in Epithelial Tissue Can Be Reversed by Coenzyme Q_10_

**DOI:** 10.1155/2018/6354680

**Published:** 2018-09-05

**Authors:** Daniel Schniertshauer, Daniel Gebhard, Jörg Bergemann

**Affiliations:** Department of Life Sciences, Albstadt-Sigmaringen University of Applied Sciences, 72488 Sigmaringen, Germany

## Abstract

The process of aging is characterized by the increase of age-associated disorders as well as severe diseases. Due to their role in the oxidative phosphorylation and thus the production of ATP which is crucial for many cellular processes, one reason for this could be found in the mitochondria. The accumulation of reactive oxygen species damaged mitochondrial DNA and proteins can induce mitochondrial dysfunction within the electron transport chain. According to the “mitochondrial theory of aging,” understanding the impact of harmful external influences on mitochondrial function is therefore essential for a better view on aging in general, but the measurement of mitochondrial respiration in skin cells from cell cultures cannot completely reflect the real situation in skin. Here, we describe a new method to measure the mitochondrial respiratory parameters in epithelial tissue derived from human skin biopsies using a XF24 extracellular flux analyzer to evaluate the effect of coenzyme Q10. We observed a decrease in mitochondrial respiration and ATP production with donor age corresponding to the “mitochondrial theory of aging.” For the first time *ex vivo* in human epidermis, we could show also a regeneration of mitochondrial respiratory parameters if the reduced form of coenzyme Q10, ubiquinol, was administered. In conclusion, an age-related decrease in mitochondrial respiration and ATP production was confirmed. Likewise, an increase in the respiratory parameters by the addition of coenzyme Q10 could also be shown. The fact that there is a significant effect of administered coenzyme Q10 on the respiratory parameters leads to the assumption that this is mainly caused by an increase in the electron transport chain. This method offers the possibility of testing age-dependent effects of various substances and their influence on the mitochondrial respiration parameters in human epithelial tissue.

## 1. Introduction

Oxidative phosphorylation is the main metabolic pathway for the production of cellular energy. It is located at the inner mitochondrial membrane where a series of redox reactions convert dietary energy from organic acids and fats into utilizable energy in the form of ATP. This close proximity to reactive oxygen species (ROS) generation sites makes mitochondrial DNA (mtDNA) vulnerable to oxidative damages. Lack of protective histones and limited repair processes in mitochondria may thus promote a high mutation rate of mtDNA [1, 2]. Those mutations have been identified as an important factor in aging processes as well as pathogenesis of inherited and age-related diseases [3]. Being the main site of ROS generation, mitochondria are thought to be part of a process in which continuous accumulation of ROS damaged mtDNA and proteins induces electron transport chain (ETC) dysfunction, which in turn leads to an enhanced ROS production. According to the “mitochondrial theory of aging,” this vicious cycle is a major cause for cellular aging, tissue dysfunction, and degeneration [4]. Indeed, ROS present a mutagenic exposure for (mt)DNA throughout the whole life span of an organism which have the potency to damage all sorts of cellular structures such as proteins, lipid membranes, or DNA [5, 6]. Damaged proteins have been shown to bind to mitochondria and disturb their function [7]. Oxidation of nucleic acids in DNA is the most problematic consequence of oxidative stress. ROS can cause single- and double-strand breaks, oxidation of DNA bases (e.g. 8-oxo-7,8-dihydroguanine (8-oxoG)), and DNA-protein interactions, among others [7, 8]. In addition to the harmful properties, ROS are essential for important physiological functions such as signal transduction or the defense against viruses and bacteria. Because of their crucial function and their role in ROS metabolism, mitochondria play a central role in most aging hypotheses. The “maximum metabolic slope theory” by Prinzinger suggests a correlation between life span and energy production [9]. It postulates that aging is a consequence of a limited capacity of mitochondria to produce energy. The “defective powerhouse model of premature skin aging” by Krutmann and Schroeder combines UV-induced mtDNA mutations and infrared-induced ETC dysfunction as corporate elicitors of mitochondrial dysfunction [6]. Aging is a continuous, multifaceted process. It is a complex combination of (mt)DNA mutation, oxidative stress, improper degradation of dysfunctional proteins and organelles and their subsequent accumulation in the cell, impairment of cellular metabolism, mitochondrial dysfunction, and compromised energy production. Even though aging has been studied extensively, it is still a matter of controversy whether observed cellular changes accompanied by aging are the cause for aging or its consequence. To break the process of ROS, DNA damage, and aging, the body tries to counteract with natural substances such as (coenzyme Q10) CoQ10, among others. CoQ10 is well characterized as intrinsic part of the ETC for its role in electron transport in mitochondrial respiratory chain between complexes I/II and III and the connected proton translocation from mitochondrial matrix to intermembrane space. However, CoQ10 is not only present in mitochondrial but all membranes throughout the cell [1]. Thereby, CoQ10 plays a pivotal role in various cellular processes such as cellular transport, regulation of cytosolic NAD+/NADH ratio, cell growth and differentiation, as well as degradation of cellular compounds. CoQ10 is a potent antioxidant and the only membrane-located antioxidant synthesized endogenously [1]. It prevents lipid peroxidation and efficiently protects DNA bases and proteins during oxidative stress, which is particularly important in the case of mtDNA [10, 11]. CoQ10 treatment was shown to induce increased activity of antioxidative enzymes such as superoxide dismutase and glutathione peroxidase [12]. CoQ10 is also involved in the prevention of mitochondrial ROS formation as an obligatory cofactor for mitochondrial uncoupling proteins [13, 14]. Activation of those proteins leads to uncoupling of oxidative phosphorylation and thereby reduces mitochondrial ROS generation. Regarding the versatile effects of CoQ10 on cell metabolism, it is no surprise that CoQ10 has been shown to be a beneficial therapeutic in various age-related and degenerative conditions such as neurodegeneration, mitochondrial myopathies, age-related macular degeneration, and cardiovascular diseases [15–18]. In all those disease patterns, low levels of endogenous CoQ10 in comparison with healthy subjects have been reported. Also aging has been shown to be accompanied by reduced CoQ10 levels in the body [10]. In this study, we examined the influence of CoQ10 on mitochondrial respiratory parameters *ex vivo* in epithelial tissue derived from human skin biopsies. To do so, we analyzed the mitochondrial respiration profile after treatment with or without CoQ10. We therefore established a Seahorse XF analyzer method to measure the mitochondrial respiration *ex vivo* directly in epidermis. We observed a decrease in mitochondrial respiration and ATP production with donor age corresponding to the “mitochondrial theory of aging” that says that the impairment of DNA repair and mitochondrial function might build a vicious cycle, contributing to the generally observed decrease in DNA repair capacity and mitochondrial function with age, and thereby accelerating (skin) aging and (skin) cancer development. We observed also a regeneration of mitochondrial respiratory parameters if the reduced form of CoQ10, ubiquinol, was administered.

## 2. Materials and Methods

### 2.1. Cell Culture

All experiments were conducted in accordance with the Declaration of Helsinki and approved by the Ethics Commission of the State Medical Association of Baden-Württemberg, Germany (187-03). Patients were informed in advance and gave their written consent to the use of their samples. Experiments were carried out with skin biopsies from donors of different ages, which were received from the Kreiskrankenhaus Sigmaringen, general surgery unit, or from Aesthetic Perfection Lake Constance, plastic surgery unit, Germany. The profiles of the donors are summarized in Table 1. Depending on the size of the biopsies, they have been used either for experiments with CoQ10 and placebo treatment or for untreated measured. After surgery, biopsies were kept in PBS at 4°C for a maximum of 24 h. The next day initially, the biopsies were washed with 70% isopropanol and then with PBS. After removal of fat and connective tissue adherent to the dermis, the biopsies were cut and incubated with Dispase II (Roche Diagnostics, Indianapolis, IN, United States) at 4°C for 16 h in keratinocyte growth medium (Lonza, Walkersville, MD, United States). Afterwards, epidermis and dermis were separated.

### 2.2. CoQ10 Supplementation

In order to investigate the effect of CoQ10 on the mitochondrial respiration parameters in epidermis, we applied an ubiquinol formulation (QuinoMit Q10 fluid; MSE Pharmazeutika GmbH, Bad Homburg, Germany). CoQ10 was always freshly weighted, diluted with ultrapure water (Millipore), and supplemented to the overnight-separation media with Dispase II. CoQ10 placebo formulation lacking CoQ10 (carrier control) was administrated exactly as described for QuinoMit. Preliminary studies with different CoQ10 concentrations showed high potency at a concentration of 100 *µ*M CoQ10. Therefore, all experiments were performed using 100 *µ*M CoQ10.

### 2.3. Mitochondrial Respiration Measurements

**Figure 1:**
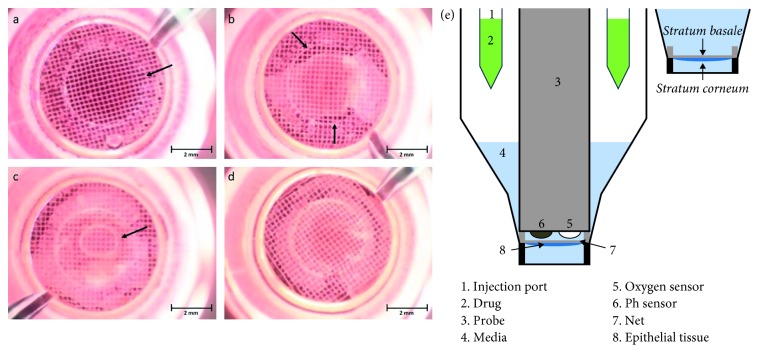
Microscopic image of an epidermis in an XF24 islet capture microplate. The epidermis was applied as flat as possible with the basal side on the net and then placed in the multiwell plate. The measurement of oxygen consumption takes place within the ring marked with the arrow (a). It was especially critical to ensure after detachment of the epidermis from the dermis that the basal side shows to the net. In addition, within the ring in the center of the net, there should be no holes (b) or air bubbles (c). Normalization of the obtained data takes place over the surface within the inner ring of the net (d). Schematic setup of the XF24 measuring system with an islet capture microplate (e).

To get a mitochondrial respiration profile, epidermis was measured with a Seahorse Bioscience XF24 Extracellular Flux Analyzer (Agilent Technologies, Santa Clara, CA, United States). XF Flux-Plates (sensor cartridge) were incubated overnight at 37°C in calibrant solution. For each treatment, a minimum of three wells were analyzed in parallel. 16 hours prior to measurement, biopsies were treated with or without 100 *µ*M CoQ10 or CoQ10 carrier control and incubated at 4°C. To measure the change of oxygen consumption rate (OCR (pmol/min)) and pH in medium surrounding the epidermis, XF24 islet capture microplates (Agilent Technologies, Santa Clara, CA, United States) were used. In these plates, small nets can be fixed to the plate bottom. To measure the oxygen consumption of the vital layer of the epidermis (stratum basale), epidermis and dermis were enzymatically separated and the epidermis placed with the vital—basal—side on the net. This orientation was essential for the measurement because the facing side of the net gets detected by the sensor. The small nets were then fixed in the microplates. It was especially critical to ensure after detachment of the epidermis from the dermis that the basal side shows to the net. In addition, within the ring in the center of the net (Figure 1), there should be no holes (Figure 1) or air bubbles (Figure 1). Samples are then incubated for 30 min in 450 *µ*L unbuffered assay medium (D7777, pH 7.4; Sigma-Aldrich, St. Louis, MO) at 37°C without CO_2_ for 1 h. During this 1 h preincubation phase, the Flux-Plate was loaded with the stress reagents (XF Cell Mito Stress Test Kit, Agilent Technologies) and instrument calibration started. Oligomycin, carbonyl cyanide-ptrifluoromethoxyphenylhydrazone (FCCP), and rotenone combined with antimycin A were all prepared to result in a 4 *µ*M final concentration after injection. After the insertion of the microplate, a 15 min equilibration phase was implemented before measurements. First, the initial respiration (basal response) was measured. Then, the active ingredients in the three ports were injected consecutively to determine the effect of each compound. This makes it possible to determine the efficiency of the individual complexes within the respiratory chain. Oligomycin blocks the *F*_0_ subunit of the ATP synthase. The decrease in respiration can thus be regarded as a measure of the ATP production of the mitochondria. The decoupling of the respiratory chain by the protonophore FCCP allows the maximal respiration to be determined independently of the proton gradient. Finally, complexes I and II are blocked by the addition of rotenone and antimycin and thus the mitochondrial respiration. The remaining oxygen consumption is thus attributable to the nonmitochondrial respiration. The measurement of the initial phase and after addition of FCCP was done in three all other phases in six cycles. Each cycle involved 4 min mixing, 2 min waiting, and 3 min oxygen consumption rate (OCR) measurement. OCR was reported in the unit of pM·min^−1^. Normalization of the obtained data takes place over the surface within the inner ring of the net (Figure 1). The difference between basal response (oxygen consumption of the third cycle) and the lowest oxygen consumption after the injection of rotenone and antimycin A gave information about the mitochondrial respiration. ATP-linked respiration was calculated from the rate of respiration inhibition by oligomycin. The maximal response was represented by the maximum respiration after FCCP injection. Proton leak was calculated by the subtraction of the lowest OCR signal after the injection of rotenone and antimycin A from the OCR signal after the addition of oligomycin.

### 2.4. Statistical Analysis

Statistical evaluation was performed using Prism 5.03 (GraphPad Software, Inc.). Values are presented as mean ± SEM or individual values. Comparison between multiple groups was performed by one-way ANOVA with Dunnett's multiple comparison test. Comparison between two columns was done by *t*-test. For correlation with age, linear regression was used. Statistical significance was defined as *p* < 0.05.

## 3. Results

### 3.1. Mitochondrial Respiration in Epithelial Tissue Depends on Donor Age

**Figure 2:**
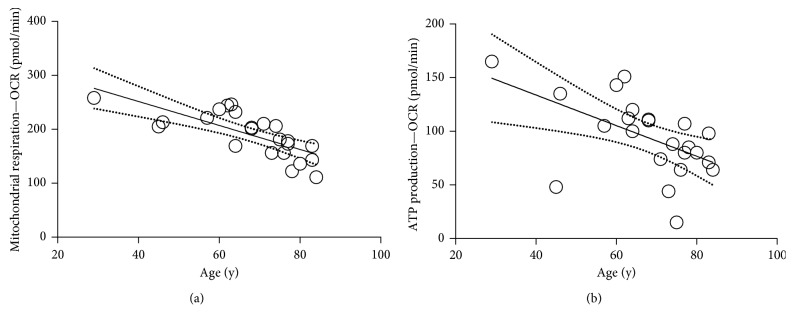
Mitochondrial respiration (a) and ATP production (b) in epithelial tissue depend on donor age. 16 hours prior to measurement, purified biopsies were stored overnight at 4°C in Dispase II. On the next day, the mitochondrial respiration of the separated epidermis was analyzed with an XF analyzer (*n* = 23, linear regression with 95% confidence interval, *R*^2^ mitochondrial respiration = 0.5487/*p* ≤ 0.0001, ATP production = 0.2945/*p* ≤ 0.01, deviation from zero = significant).

In this study, we investigated the effect of CoQ10 on respiratory parameters in epidermal biopsies of donors of various ages. First, we analyzed the mitochondrial respiration and ATP production as a function of age (Figure 2). To do so, we measured the respiration profile with a Seahorse XF analyzer. As shown, both the mitochondrial respiration and ATP production in the measured donor collective (Table 1) decrease drastically with increasing age. This results in a reduction of mitochondrial respiration by an average of approximately 10 percent per decade.

### 3.2. Effect of CoQ10 on Respiratory Parameters in Epithelial Tissue

**Figure 3:**
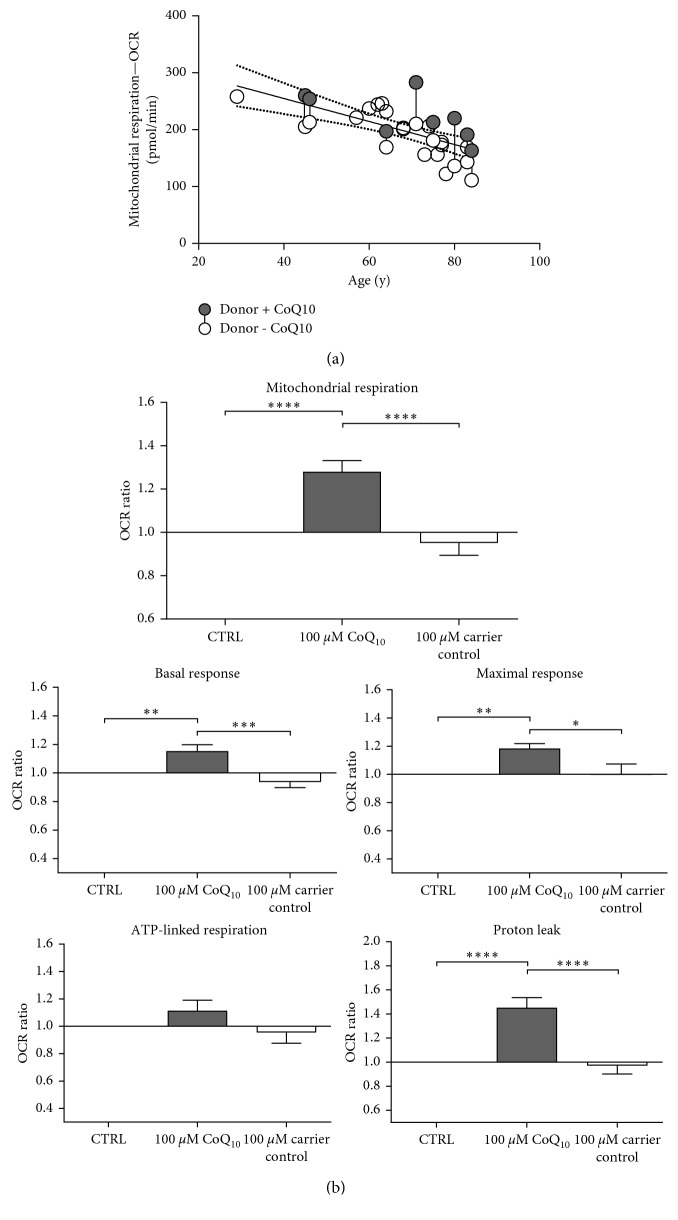
Effect of CoQ10 on respiratory parameters in epithelial tissue. 16 hours prior to measurement, purified biopsies were stored overnight at 4°C in Dispase II. During incubation with Dispase II, the samples were treated with or without 100 *µ*M CoQ10 or CoQ10 carrier control. On the next day, the respiration profile of the separated dermis was analyzed with an XF analyzer. White circles represent untreated samples. CoQ10-treated samples are shown in dark gray circles. Connected circles represent the same donor. ((a) *n* = 23, linear regression with 95% confidence interval, *R*^2^ = 0.4354/*p* ≤ 0.0001, deviation from zero = significant; (b) *n* = 8, mean ± SEM, one-way ANOVA with Dunnett's multiple comparison test versus control and CoQ10 carrier control, *∗∗∗∗p* < 0.0001). Control cells correspond to 1.0. OCR = oxygen consumption rate.

In the next experiment, the impact of CoQ10 and CoQ10 carrier control treatment on respiratory parameters was examined. Therefore, 16 hours prior to measurement, biopsies were treated with or without 100 *µ*M CoQ10 or CoQ10 carrier control and incubated at 4°C. Due to the small size of many biopsies, treatment with CoQ10 and carrier control could not be performed on all samples obtained. Preliminary studies with different CoQ10 concentrations showed high potency at a concentration of 100 *µ*M CoQ10. Therefore, all experiments were performed using 100 *µ*M CoQ10. To measure the change of OCR and pH in medium surrounding the epidermis, XF24 islet capture microplates (Agilent Technologies, Santa Clara, CA, United States) were used. CoQ10 supplementation induced an increase in mitochondrial respiration (Figure 3(a)) and also increased almost all key respiratory parameters, while there was no effect of CoQ10 carrier control (Figure 3(b)). A significant increase in basal response, maximal response, mitochondrial respiration, and proton leak with 100 *µ*M CoQ10 was observed. ATP-linked respiration was also increased but not significantly. Carrier control-treated epidermis cells showed no significantly higher respiration parameters. Therefore, CoQ10 treatment had a positive effect on respiratory parameters in epithelial tissue.

### 3.3. Influence of Sampling Location of Biopsies on Mitochondrial Respiration

**Figure 4:**
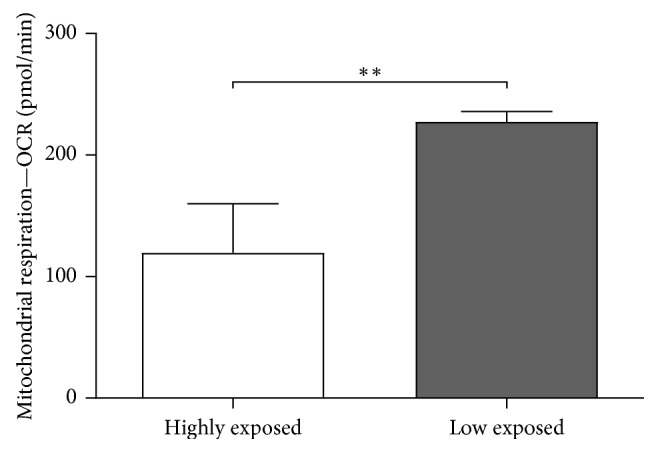
Influence of sampling location of biopsies on mitochondrial respiration. 16 hours prior to measurement, purified biopsies were stored overnight at 4°C in Dispase II. On the next day, the respiration profile of the separated dermis was analyzed with an XF analyzer. The range of age in both groups (HE and LE) was between 29 and 77 years (*n* = 6 highly exposed, *n* = 6 low exposed, linear regression, mean ± SEM, *t*-test, *∗∗p* < 0.05).

In order to exclude a photoaging effect on the values obtained, the mitochondrial respiration of biopsies from body regions with a high UV exposure (HE) (e.g., eyelids) and biopsies from low UV-exposed body regions (LE) (e.g., abdomen) were randomly compared (Figure 4). For this, we measured the mitochondrial respiration profile with a Seahorse XF analyzer. Samples with a high UV exposure showed a significantly lower mitochondrial respiration on average than samples with a low UV exposure. These samples (HE) were not included in the evaluation of age dependence.

## 4. Discussion

The term aging is often defined as the accumulation of damage and harmful changes within the cell over time, which can lead to an increased incidence of disease but in any case leads to death. The exact relationship between the accumulation of this damage and aging itself is not fully understood. It is assumed that a reduction of CoQ10 biosynthesis during aging as well as in age-related diseases plays a significant role in here [19, 20]. In addition, mitochondria and their role in energy production are increasingly becoming the main players in the process of aging [21, 22]. Therefore, CoQ10 has become a popular study object in recent years and the increasing evidence for its diverse positive properties has led to an increasing use of CoQ10 in mitochondrial medicine in the therapy of various (degenerative) diseases.

This study investigated the effect of CoQ10 on the mitochondrial respiratory parameters with increasing age directly in epithelial tissue, isolated from human skin biopsies. We observed a decrease in mitochondrial respiration as well as in ATP production with donor age and a regeneration of mitochondrial respiratory parameters by the addition of CoQ10. The observed decrease in mitochondrial respiration and ATP production with donor age in the first experiment, approximately ten percent per decade, corresponds to the “mitochondrial theory of aging” that says that the impairment of DNA repair and mitochondrial function might build a vicious cycle, contributing to the generally observed decrease in DNA repair capacity and mitochondrial function with age [18, 23–26]. With increasing age, the number of DNA damage in mitochondria increases. If these damages are not repaired, mutations occur. These mutations can lead to age-associated diseases, but at least to a loss of capacity in the ETC [9]. The results of this study correlate with the “maximum metabolic slope theory” by Prinzinger [9] that suggests a correlation between life span and energy production. According to this, aging is a consequence of the fact that the mitochondria can no longer provide sufficient energy. A decrease in mitochondrial respiration and ATP production with increasing age is a clear proof of this. In a further experiment, we could show that treatment with CoQ10 leads to a significant increase in most mitochondrial respiratory parameters in human epithelial tissue. Preliminary experiments tested cell viability and proliferation of fibroblasts using different CoQ10 concentrations in a range of 0–1000 *μ*M. The experiments showed no toxic or antiproliferative effect of CoQ10 within this range. Furthermore, the safety of CoQ10 is well documented in rats and humans [27, 28]. The dosages chosen complied with dosages used in other in vitro studies [29–32]. 100 *μ*M showed a significant change in most respiratory parameters and was therefore chosen for this study. CoQ10 carrier control in the same concentration as CoQ10 revealed no significant impact of the additives contained in QuinoMit Q10 fluid. Therefore, all results were related to CoQ10 administration. The age-related decrease in mitochondrial respiration was diminished by CoQ10 treatment. It could be shown that almost all key respiratory parameters were increased. An increase can be attributed to an enhanced ETC. Estornell et al. reported a correlation between CoQ10 concentration and respiratory rate which led to the assumption that physiological CoQ10 concentrations do not saturate the respiratory chain [33, 34]. Thus, CoQ10 administration was expected to induce an increase of mitochondrial respiration. These assumptions could be confirmed. It is assumed that the addition of CoQ10 results in improved electron transport and thus an increase in respiratory parameters. The “free radical theory of aging” by Harman says that the accumulation of mitochondrial damage leads to physiological dysfunctions and finally to pathological diseases [4]. It has been suggested that the mitochondrial respiratory chain become “leaky,” releasing more ROS [35]. For the role of mitochondrial dysfunction and ROS release in age-related diseases, there is abundant evidence [36–38]. Also the accumulation of oxidative damage with age is proven [39].

We suppose that exogenously added CoQ10 acts as an “electron clamp” and thus enhances the electron transport between complexes I/II and III in the mitochondrial respiratory chain which has become “leaky” over the years. This could explain the increased mitochondrial respiration after CoQ10 treatment. On the other hand, the production of ROS could thereby be reduced and thus cellular damage, aging, and disease be attenuated.

## 5. Conclusions

In conclusion, an age-related decrease in mitochondrial respiration and ATP production in accordance with the mitochondrial theory of aging was confirmed. Likewise, an increase in the respiratory parameters by the addition of CoQ10 could also be shown. The fact that there is a significant effect of administered CoQ10 on the respiratory parameters leads to the assumption that this is mainly caused by an increase in the ETC. Low levels of endogenous CoQ10 have been implicated in various age-related and chronic diseases. Often, mitochondrial dysfunction and/or low antioxidant activity play an important role. The ability of CoQ10 to improve the electron transport chain and its antioxidant potential makes it an important therapeutic agent for influencing the development of some age-associated diseases and the aging process itself. Despite these facts, further studies performed on humans and in advance on human tissue are needed to better understand the promising effects of CoQ10 and before CoQ10 can be considered as an effective antiaging therapy. The mitochondrial respiration assay we established for this study offers the possibility of testing age-dependent effects of various substances and their influence on the mitochondrial respiration parameters in human epithelial tissue.

## Figures and Tables

**Table 1:** Summary of all donors.

Donor	Age (y)	Sex	Smoking/nonsmoking	Body site
1	29	F	s	Femoral
2	35	F	ns	Upper eyelids
3	45	M	ns	Foreskin
4	46	M	ns	Abdomen
5	46	F	ns	Upper eyelids
6	54	F	ns	Upper eyelids
7	55	F	ns	Upper eyelids
8	57	F	ns	Abdomen
9	57	M	ns	Upper eyelids
10	60	M	ns	Abdomen
11	62	F	ns	Abdomen
12	63	M	ns	Abdomen
13	64	M	s	Abdomen
14	64	M	s	Abdomen
15	68	F	ns	Abdomen
16	68	M	ns	Abdomen
17	71	M	ns	Abdomen
18	73	F	ns	Abdomen
19	74	F	s	Abdomen
20	75	F	s	Abdomen
21	76	M	ns	Abdomen
22	77	M	s	Abdomen
23	77	M	ns	Upper eyelids
24	77	M	s	Abdomen
25	78	M	ns	Abdomen
26	80	F	ns	Abdomen
27	83	M	ns	Abdomen
28	83	F	ns	Abdomen
29	84	M	ns	Abdomen

F: female; M: male; ns: nonsmoking; s: smoking.

## Data Availability

Data and materials related to this work are available upon request.
